# Expression of Human Epidermal Growth Factor Receptor-2 in Resected Rectal Cancer

**DOI:** 10.1097/MD.0000000000002106

**Published:** 2015-10-30

**Authors:** Xiangjiao Meng, Zhaoqin Huang, Jian Di, Dianbin Mu, Yawei Wang, Xianguang Zhao, Hanxi Zhao, Wanqi Zhu, Xiaolin Li, Lingling Kong, Ligang Xing

**Affiliations:** From the Department of Radiation Oncology (XM, JD, XZ, HZ, WZ, XL, LK, LX), Shandong Cancer Hospital and Institute; Department of Radiology (ZH), Provincial Hospital Affiliated to Shandong University; Department of Pathology (DM), Shandong Cancer Hospital and Institute; and Department of Chemotherapy (YW), Qilu Hospital, Shandong University, Jinan, Shandong Province, China.

## Abstract

The addition of trastuzumab to chemotherapy was demonstrated to be beneficial for advanced human epidermal growth factor receptor-2 (HER-2) positive gastric cancer. However, the HER-2 status of rectal cancer remains uncertain. This study aimed to determine the HER-2 expression in a large multicenter cohort of rectal cancer patients. The clinical and pathological features of 717 patients were retrospectively reviewed. All the patients were diagnosed with primary rectal adenocarcinoma without distant metastasis and took surgery directly without any preoperative anticancer treatment. HER-2 status was assessed on resected samples. A total of 99 cases with IHC3+ and 16 cases with IHC 2+ plus gene amplification were determined as HER-2 positive. 22.6% of HER-2 positive patients had local recurrence, whereas 16.9% of HER-2 negative patients did (*P* = 0.146). HER-2 positive tumors were more likely to have distant metastasis (*P* = 0.007). Univariate analysis revealed that pathological tumor stage, pathological node stage, positive margin, and lymphovascular invasion were significantly correlated with 5-year disease-free survival (DFS) and 5-year overall survival (OS). The patients with >10 dissected lymph nodes showed significantly longer OS (*P* = 0.045) but not DFS (*P* = 0.054). HER-2 negative patients had significantly better 5-year DFS (*P* < 0.001) and 5-year OS (*P* = 0.013) than those of the HER-2 positive patients. In the subgroup analysis for the early rectal cancer and locally advanced rectal cancer, HER-2 was also a poor predictor for survival. Multivariate analysis revealed that HER-2 was an independent prognostic factor for 5-year DFS (hazard ratio [HR] = 1.919, 95% confidence interval [CI] 1.415–2.605, *P* < 0.001) and for 5-year OS (HR = 1.549, 95% CI 1.097–2.186, *P* = 0.013). When the treatment was included in the analysis for locally advanced patients, HER-2 was a prognostic factor for 5-year DFS (*P* = 0.001) but not for 5-year OS (*P* = 0.106). This study confirmed that HER-2 was expressed in a part of patients with rectal cancers and might be used as a negative predictor. The results may support the trials to assess the efficacy of trastuzumab in treating HER-2 positive rectal cancer patients.

## INTRODUCTION

Rectal cancer is one of the leading causes of cancer-related deaths in the world.^1^ Compared with postoperative chemoradiotherapy (CRT), preoperative CRT was demonstrated to improve local control and decrease toxicity.^2^ Therefore, preoperative CRT followed by surgery has been implemented as a standard treatment strategy for the locally advanced rectal cancer (LARC).^2–4^ However, both the disease-free survival (DFS) and overall survival (OS) were similar between preoperative and postoperative CRT.^2^ A large number of patients take surgery directly with or without postoperative treatment, which depend on pathological results. Pathological tumor staging is used to predict survival and guide the postoperative therapy. However, there is uncertainty concerning the difference in prognosis with patients who have the same pathological stage. Thus, it is helpful to find a suitable biomarker to predict the prognosis for patients undergoing surgery directly and identify the patients who need postoperative treatment. It is also interesting to explore such biomarkers as targets for anticancer treatment to improve patient's survival.

The human epidermal growth factor receptor-2 (HER-2) is a 185 kDa transmembrane oncoprotein belonging to the human epidermal growth factor family.^5^ Its tyrosine kinase activity alone plays important roles in cell proliferation, differentiation, migration, and survival.^6,7^ Originally, HER-2 was widely studied in breast cancer, and its overexpression is typically correlated with more aggressive activity and a worse prognosis.^8,9^ As a successful target for trastuzumab in treatment of breast cancer, HER-2 status in other solid tumors has also been analyzed as well. HER-2 expression has been detected in lung cancer,^10^ type I endometrial carcinoma,^11^ esophageal cancer,^12^ and was demonstrated as a prognostic factor.^10–12^

In gastric cancer, HER-2 overexpression was observed to be associated with aggressiveness and poor outcomes.^7,13^ The study on trastuzumab for gastric cancer (ToGA trial) demonstrated that the addition of trastuzumab to chemotherapy was beneficial for advanced HER-2 positive gastric cancer.^14^ Trastuzumab has been approved for the treatment of patients with HER-2 positive metastatic adenocarcinoma of the stomach or gastroesophageal junction.^15^

For both rectal and gastric cancers in gastrointestinal tract, their biological characters are similar in many ways, that is they are adenocarcinoma with high rate of liver metastasis and effective chemotherapy regimen.^16,17^ Given the predictive value of HER-2 protein with response to trastuzumab in gastric cancer, it is interesting to study the HER-2 positivity in rectal cancer.

In our previous study, HER-2 status was determined in pretreatment biopsies in 119 LARC patients. HER-2 positivity was a negative prognostic factor for LARC patients who took neoadjuvant CRT.^18^ However, our previous study was limited by small sample size and only patients with preoperative CRT included. The frequency and the prognosis role of HER-2 in rectal cancer still remain controversial. Thus, this study aimed to determine the HER-2 status and its predicting value of clinical outcome in a large multicenter cohort of rectal cancer patients undergoing surgery directly.

## MATERIALS AND METHODS

### Patients

The clinical and pathological features of patients were reviewed retrospectively for the purpose of the study from January 2007 to December 2009 in Shandong Cancer Hospital and Institute, Provincial Hospital Affiliated to Shandong University, and Qilu Hospital, Shandong University. This retrospective research was approved by the Institutional Ethics Committee. All the patients were diagnosed with primary rectal adenocarcinoma. Preoperative diagnosis was established by a series of examinations, including physical examination, peripheral blood cell count, hepatic function, renal function, chest x ray or computed tomography (CT), contrast-enhanced CT, and/or magnetic resonance imaging (MRI) of the abdomen and pelvis. The patients took surgery directly without any preoperative anticancer treatment.

Patients who had a distant metastasis diagnosed during the initial operation, or synchronous tumors, or a history of other malignant tumors, or without following-up materials were excluded. Tumor, node, and metastasis stages were assessed according to the American Joint Committee on Cancer.^19^ Epidemiological data, including age, sex, and tumor distance from the anal verge, were obtained from preoperative medical records. The data for the tumor, such as T-stage, lymph node metastatic status, differentiation, lymphovascular invasion (LVI), and margin, were assessed according to pathology diagnosis.

### Postoperative Follow-Up

Follow-up examinations were routinely taken after treatment. The patients took colonoscopy at 1 year after surgery and then once at the interval of 2 or 3 years. The contrast-enhanced CT or MRI of abdomen and pelvis were performed every 6 months. Physical examinations, blood examination, stool routine test, and so on were performed once at every 3 months for the first 2 years and then at every 6 months. For survival analysis, DFS was defined as the time from the date of surgery to any evidence of local recurrence or distant metastasis. OS was defined as the interval between the date of surgery and the date of death or the censored date of last follow-up. The median follow-up time was 60 months for surviving patients.

### Assessment of HER-2 Status by Immunohistochemistry (IHC)

The HER-2 expression was performed on 5-μm-thick slices of resected samples according to the proposal by Meng et al.^18^ After deparaffinization and rehydration, the tumor antigen was retrieved. Following the process of nonspecific binding, primary monoclonal rabbit anti-human HER-2 antibody was incubated at 37°C for 60 minutes (Abcam, Cambridge, UK). Then the sections were stained with secondary goat anti-rabbit antibody (Beijing Zhongshan Golden Bridge Biotechnology Company, China) at 37°C for 15 minutes. The slices was visualized and counterstained by 3,3’-diaminobenzidine and hematoxylin, respectively. After dehydration, the stained slices were visualized under a microscope (Olympus, Tokyo, Japan).

The intensity of staining and percentage of stained cells were used to evaluate the HER-2 expression according to ToGA trial^14^: IHC 0 (absent), no staining; IHC1+ (low), faint intensity in 10% or more; IHC2+ (moderate), moderate intensity in 10% or more; IHC3+ (high), strong intensity in 10% or more of cancer cells.

### Assessment of HER-2 Gene Amplification

HER-2 gene amplification was performed to confirm HER-2 expression in all the samples with an IHC score of 2+ by the fluorescence in situ hybridization (FISH) using the PathVysion HER-2 DNA Probe Kit/centromere (CEP) 17 probes (Abbott Molecular, Des Plaines, IL).^14,18^ Briefly, 5-μm-thick tissue slices were deparaffinized and rehydrated consecutively with distilled water through a graded series of ethanol solutions. The specimens were fixed before assaying with the PathVysion Kit. To denature the DNA, the slices were incubated at 85°C for 5 minutes. Then 10 μL of probe mixture was applied to the target area, and a cover slip was immediately placed on the probe. Then slices were incubated at 37°C overnight in the pre-warmed humidified hybridization chamber, and then washed and counterstained. The complete tissue sections were visualized under a fluorescence microscope (Olympus). According to ToGA guidelines, a total of 60 representative nuclei from the tumor cells were scored. A specimen with a HER-2/CEP17 ratio of 2.0 or higher in tumor cells was classified as HER-2 amplification.^14^

### Statistical Analysis

Statistical Product and Service Solutions (SPSS17.0) was used for all the statistical analysis. The patients without follow-up data were excluded. The correlations of HER-2 status with clinical and pathological parameters, including age, sex, differentiation, tumor distance from anal verge, pathological tumor stage (pT), and pathological node stage (pN), were assessed using the χ^2^ test. The associations of local recurrence or distant metastasis with clinical and pathological parameters including HER-2 status were also assessed using the χ^2^ test. The impact of the HER-2 status on survival was determined using Kaplan-Meier method for the univariate analysis. Cox proportional hazards model was used for the multivariate analysis to calculate hazard ratios (HRs) and 95% confidence intervals (95% CIs). *P* value <0.05 was considered statistically significant.

## RESULTS

### Characteristics of Patients

A total of 717 patients with rectal cancer were included in this study. The clinical and pathological parameters were shown in Table 1. In brief, there were 416 males and 301 females with 344 cases being >60 years. A total of 193 cases were assessed as pathological tumor stages 1 and 2 (pT1–2), and 524 cases were assessed as pT3–4. A total of 340 patients had node metastasis. A total of 401 locally advanced patients (pT3–4 or N+) received postoperative treatment with 5-fluorouracil-based chemotherapy and concurrent radiation therapy (45–54 Gy/25–30 fractions). A total of 162 patients with LARC did not receive postoperative treatment for adverse effect of CRT or economic reasons.

**TABLE 1:**
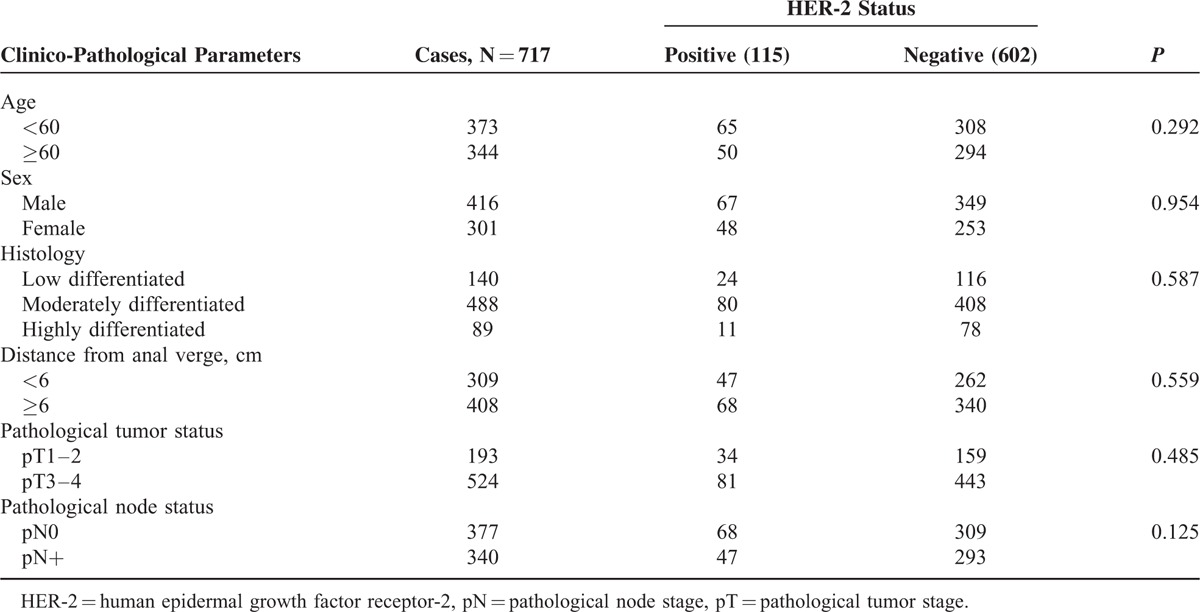
Correlations Between HER-2 Expression and Clinico-Pathological Parameters

### Correlations of HER-2 Status With Clinical and Pathological Parameters

**FIGURE 1:**
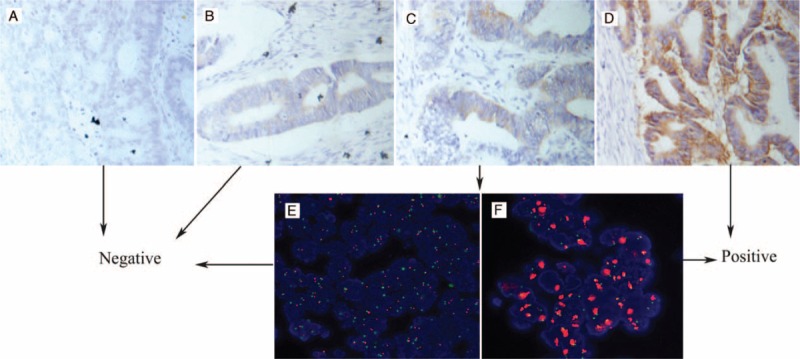
Representative immunohistochemical (IHC) staining and fluorescence in situ hybridization (FISH) for HER-2 in rectal cancer cells (400×): (A) nonstaining is observed (0); (B) faint staining is observed in >10% of tumor cells (1+); (C) moderate staining is observed in >10% of the tumor cells (2+); (D) strong staining is observed in >10% of the tumor cells (3+); (E) tumors without HER-2 amplification; and (F) tumors exhibited HER-2 amplification with an HER-2/CEP17 ratio >2.0. FISH = fluorescence in situ hybridization, HER-2 = human epidermal growth factor receptor-2, IHC = immunohistochemical.

Among all patients, 201 cases were classified as IHC score 0, 193 cases as IHC score 1+, 224 cases as IHC score 2+, and 99 cases as IHC score 3+, respectively (Fig. 1A–D). HER-2 gene amplification was detected in 16 cases of 224 patients with IHC score 2+ (Fig. 1E, F). A total of 115 cases (16%) were determined as HER-2 positive. Correlation analysis showed no significant associations of HER-2 positivity with any clinical and pathological parameters such as sex, age, differentiation, pT, and pN.

### Correlations Between Clinico-Pathological Parameters and Tumor Recurrence

The χ^2^ test was used to determine the ability of clinico-pathological parameters to predict local recurrence and distant metastasis (Table 2). Age, sex, differentiation, and anal verge distance were not observed to be correlated neither with local recurrence nor with distant metastasis. Both pT and pN showed highly significant correlations with local recurrence and distant metastasis (*P* < 0.001). Twenty-six cases out of 115 HER-2 positive patients had local recurrence, whereas only 102 cases out of 602 HER-2 negative patients did (*P* = 0.146). Compared with HER-2 negative patients, HER-2 positive tumors appeared more likely to have distant metastasis (36.5% vs 64.2%, *P* = 0.007).

**TABLE 2:**
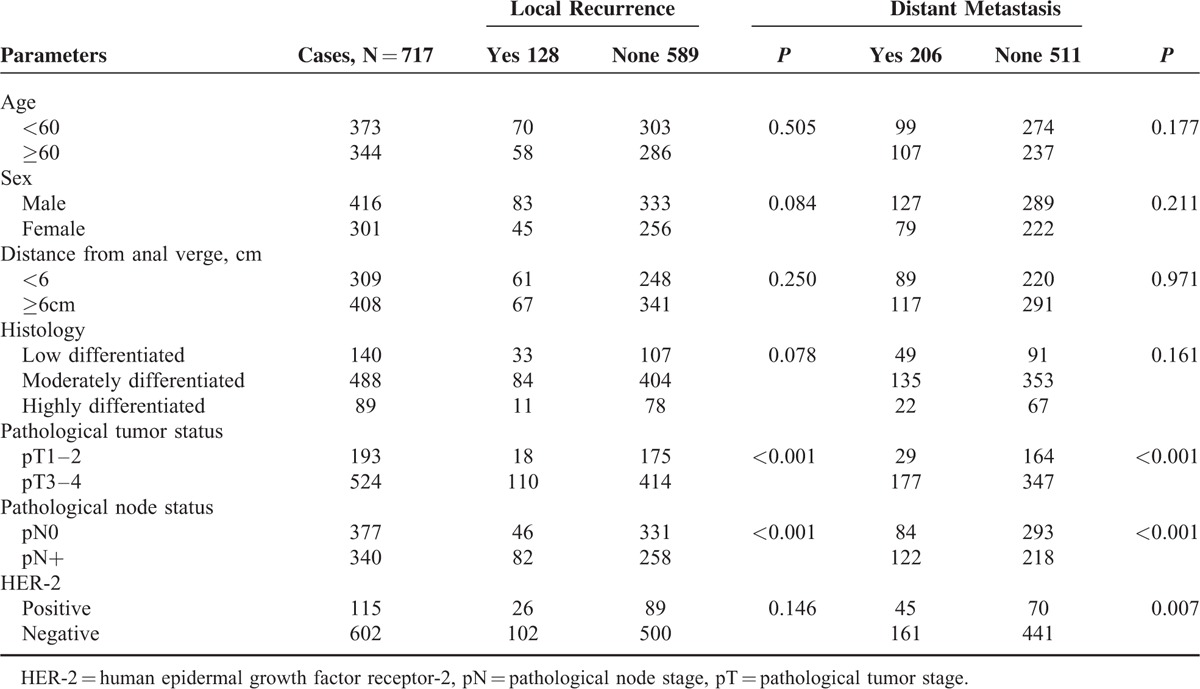
Correlations of Clinico-Pathological Parameters With Local Recurrence and Distant Metastasis

### Survival Analysis

**FIGURE 2:**
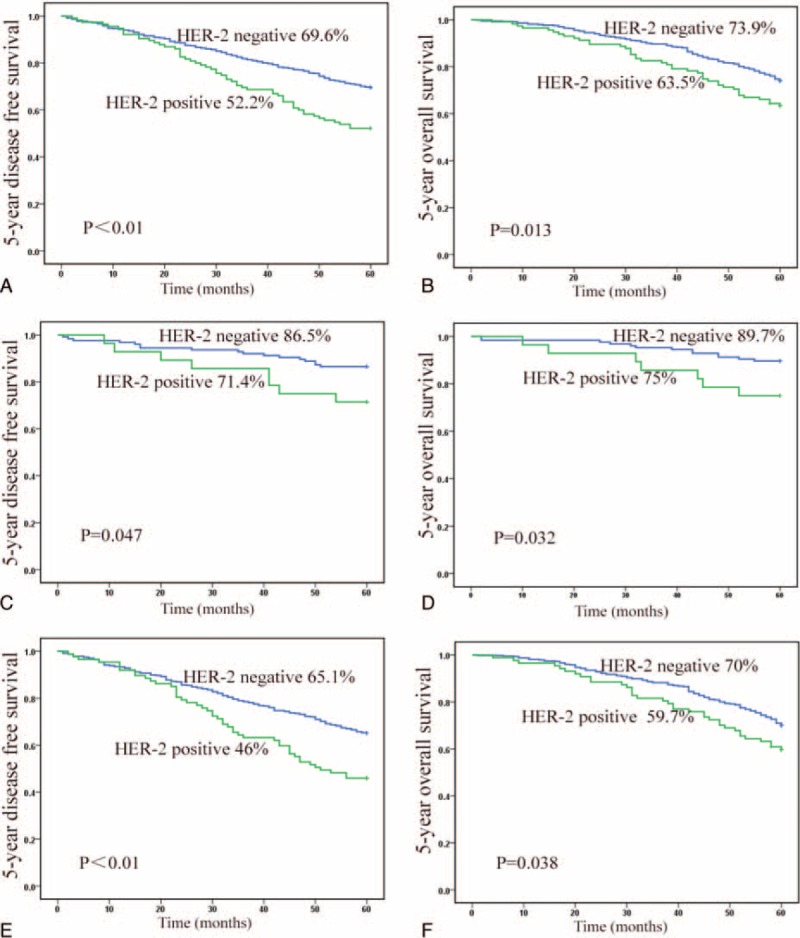
Kaplan-Meier analysis of DFS and OS rates in relation to HER-2 status: (A) HER-2 overexpression in rectal cancers is correlated with a shorter DFS curves (*P* < 0.001); (B) HER-2 overexpression in rectal cancers is correlated with a shorter OS curves (*P* = 0.013); (C) HER-2 was a poor predictor for 5-year DFS in rectal cancers at early stage (*P* = 0.047); (D) 5-year OS curves in patients with rectal cancer at early stage (*P* = 0.032); (E) HER-2 was a poor predictor for 5-year DFS in LARCs (*P* < 0.001); (F) 5-year-OS curves in patients with LARC (*P* = 0.038). DFS = disease-free survival, HER-2 = human epidermal growth factor receptor-2, LARC = locally advanced rectal cancer, OS = overall survival.

No correlations of survival with age, sex, anal verge distance, or differentiation were found (Table 3). Univariate analysis revealed that pT, pN, positive margin, and LVI were significantly correlated with 5-year DFS and 5-year OS. The patients with >10 dissected lymph nodes showed longer OS (*P* = 0.045) but not DFS (*P* = 0.054). The locally advanced patients receiving postoperative treatment showed longer survival than those without treatment. The 5-year DFS and 5-year OS of patients with HER-2 positive status were significantly shorter than those of HER-2 negative patients (52.2% vs 69.6%, *P* < 0.01 and 73.9% vs 63.5%, *P* **=** 0.013) (Fig. 2A, B). In the subgroup of early cancer (pT1,2 N0) or locally advanced cancer, the HER-2 status was also a negative predictor for both 5-year DFS and 5-year OS (*P* < 0.05) (Fig. 2C–F).

**TABLE 3:**
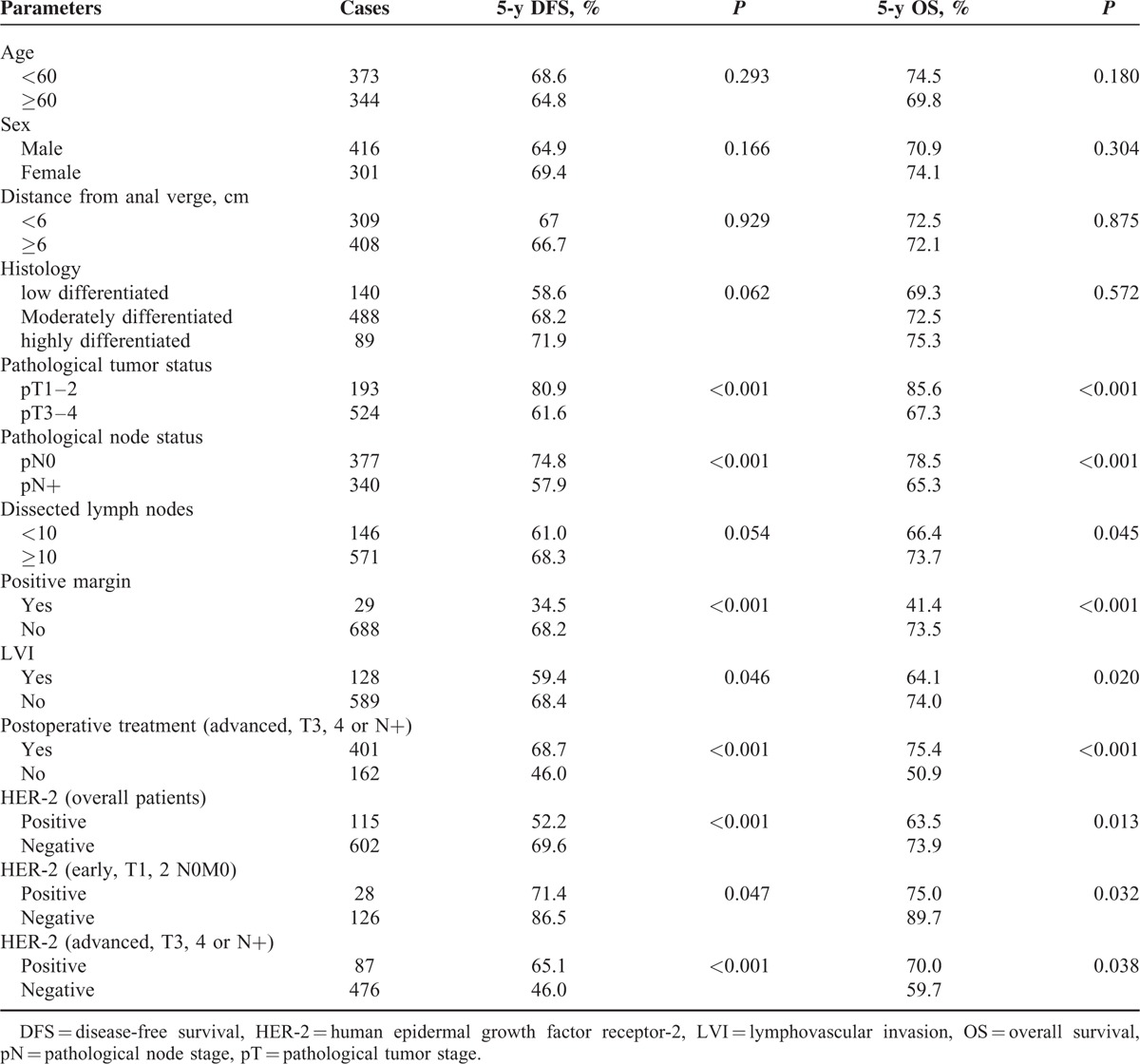
Univariate Analysis on Correlations Between Clinico-Pathological Parameters and Survival

According to the results of univariate analysis, pT, pN, HER-2, positive margin, and LVI were included in multivariate analysis (Table 4). Dissected lymph nodes were included in multivariate analysis for 5-year OS but not for DFS. Among all the patients, HER-2 was an independent prognostic factor for both 5-year DFS (HR = 1.919, 95% CI 1.415–2.605, *P* < 0.001) and 5-year OS (HR = 1.549, 95% CI 1.097–2.186, *P* = 0.013). When treatment was included in multivariate analysis for locally advanced patients, HER-2 was a prognostic factor for 5-year DFS (*P* = 0.001) but not for 5-year OS (*P* = 0.106).

**TABLE 4:**
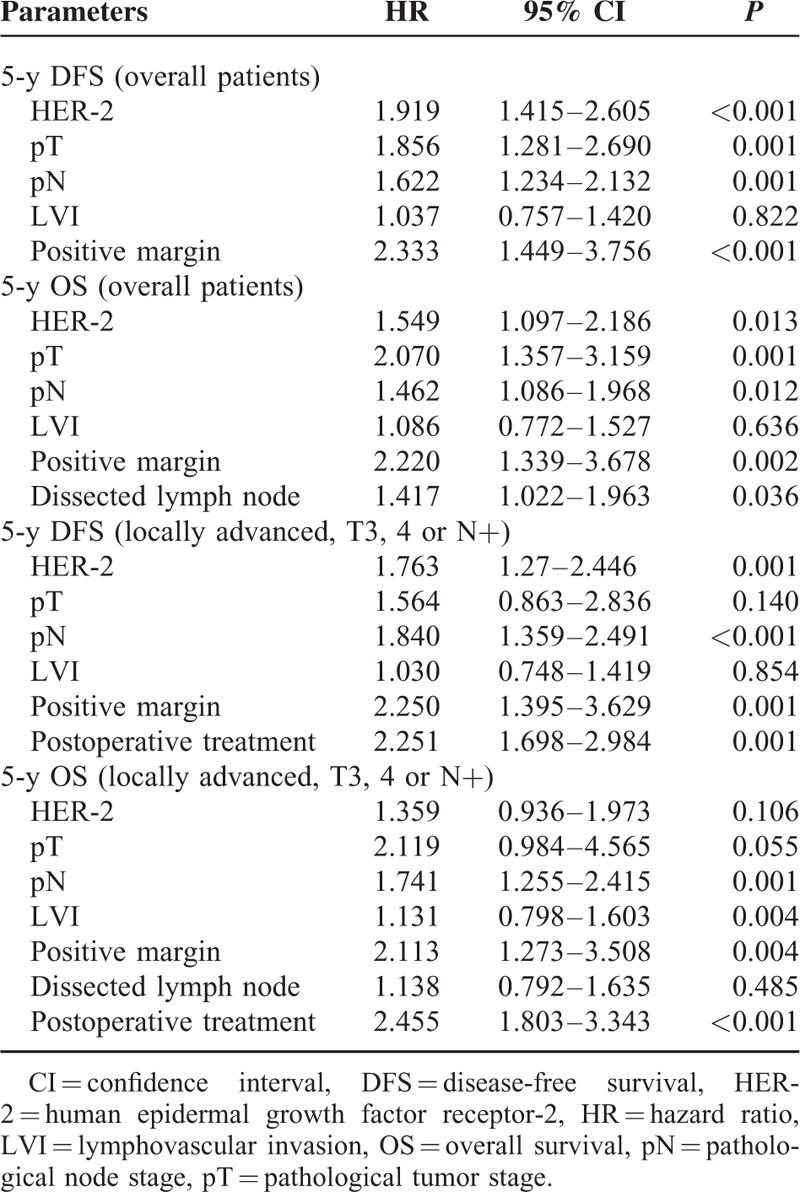
Multivariate Analysis for Survival

## DISCUSSION

As HER-2 is the target of trastuzumab, it is important to clarify the HER-2 status and its predictive value in rectal cancer. In the present study, we identified 115 HER-2 positive cases in 717 patients. The results demonstrated that HER-2 was a negative predictor for prognosis in rectal cancer.

IHC, which can be taken in all the department of pathology, is currently the most widely used method for the assessment of HER-2 expression. FISH is regarded as the “gold standard” for the detection of HER-2 amplification with high sensitivity (96.5%) and specificity (100%).^20^ Compared with breast cancer cells, gastric cancer cells exhibited more frequently incomplete membrane staining in a basolateral pattern.^21^ Thus, if adopting breast cancer scoring criteria for HER-2 status in gastric cancer would lead to lower positivity with >50% false-negative rate.^21–23^ Therefore, Hofmann et al proposed a modified HER-2 scoring system specific for gastric cancer.^14,21^ The scoring system has been proved to be reproducible, and thus has been widely adopted. Because both rectal cancer and gastric cancer in gastrointestinal tract, it is appropriate to assess the status of HER-2 according to ToGA criteria used for gastric cancer.

HER-2 status was studied as single predictive marker in this research for several reasons. First, in digestive tract tumor, HER-2 was usually assessed as a single biomarker for prognosis.^7,12,13^ Second, up to date, all biomarkers for prognosis have not been effective enough in clinical applications. Thus, it is difficult to choose one biomarker which may combine with HER-2 in prediction. Third, because the predictive value of HER-2 remains unclear in rectal cancer, the predictive value of the combination of it with other biomarkers or genes needs to be explored in the future study.

In the current study, 115 cases of 717 patients with rectal cancer were determined as HER-2 positive. The percentage of positivity was not consistent with those in previous reports. For instance, Park et al^24^ detected positive staining for the HER-2 protein in 47.4% of the 137 colorectal carcinomas (CRCs). Drebber et al^25^ found an HER-2 staining in 27% of rectal cancer. A recent study revealed a moderate HER-2 expression in 14% CRC patients and strong expression in 11% CRC patients.^26^ The discrepancy between different studies can be explained by following factors. First, the criteria used for HER-2 positivity in these studies were different; even in the study by Park et al, both IHC 2+ and IHC 3+ were considered as HER-2 positive. Second, as indicated in the previous report,^27^ there were differences in HER-2 positivity between biopsies and resected samples. Similar with previous studies,^26,27^ no significant correlations were found between HER-2 status and clinico-pathological parameters.

In the current study, 22.6% of HER-2 positive patients had local recurrence, whereas 16.9% of HER-2 negative patients did (*P* = 0.146). Although the difference was not significant, a trend can be seen that HER-2 positive patients experienced more recurrence than HER-2 negative patients did. Our data indicated that distant metastasis occurred more frequently in HER-2 positive patients (*P* = 0.007). Only a more recently published study reported that HER-2 overexpression was correlated with more distant metastasis in rectal cancer.^28^ This correlation might be attributed to the HER-2 functions in cell proliferation, adhesion, and migration.^6^ Gene expression analysis revealed that the E2F transcription factors were integral to the development and progression of HER-2 positive tumor.^29^

In the present study, the HER-2 status was analyzed in a large population of patients recruited from 3 cancer centers, and its overexpression was found to be correlated significantly with shorter 5-year DFS and 5-year OS, as revealed by both univariate analysis and multivariate analysis for all the patients. When treatment was included in multivariate analysis for LARC patients, HER-2 was a prognostic factor for 5-year DFS (*P* = 0.001) but not for 5-year OS (*P* = 0.106). The results suggested that postoperative treatment might reduce the difference in survival between HER-2 positive and HER-2 negative patients. We must cautiously understand these results because clinical trials are needed to confirm whether HER-2 positive rectal patients acquire more benefits from postoperative CRT or not.

Data with respect to the putative role of HER-2 in rectal cancer are conflicting. For instance, a study from Japan reported that OS for patients at Dukes stage B was significantly lower in the group with overexpression of cytoplasmic HER-2 than in the group without HER-2 expression.^30^ Lim et al^26^ found that the survival of patients without HER-2 expression was significantly better than that of patients with positive expression of HER-2. In our previous study with small patient population, we found that the positive HER-2 status was significantly associated with shorter 5-year DFS (*P* = 0.015) and 5-year OS (*P* = 0.026).^18^ However, some studies did not support the association between HER-2 status and poor prognosis. Based on the analysis of the pre-therapeutic biopsies, Drebber et al^25^ found that patients with a higher level of HER-2 expression showed a trend of a better DFS (*P* = 0.1) and a significant benefit in cancer-specific survival (*P* = 0.03). In another study reported from China, no significant correlation was found between HER-2 overexpression and survival.^28^ We speculated that there might be several reasons explaining the conflicting results among studies: first, the methodologies for HER-2 assessment and the criteria for positive definition varied substantially in different clinical studies. Second, the discrepancies between Asian and Western data may, at least in part, influence the results. Third, the patient inclusion criteria in previous studies were different and the number of patients was small. Furthermore, we first studied the role of HER-2 protein in rectal cancer at early stage, and demonstrated that it was also a poor prognosis predictor. The results from our current study are in accordance with those of the previous study in gastric cancer at the early stage, which demonstrated that HER-2/neu could serves as a prognostic marker to predict the risk of poor outcome (*P* = 0.005).^31^

There are some inherent limitations of this study. First, the current study was retrospective, and the tumor samples have been preserved for >5 years, which might lead to some biased results. Second, the patients with locally advanced cancer did not receive neoadjuvant treatment, the standard therapy. We must be cautious that the results may not be suitable for all rectal patients. But in fact, a lot of patients still refuse neoadjuvant treatment and the HER-2 status assessed on such resected regimens might be not affected by treatment.

In conclusion, 115 cases out of 717 rectal cancer patients undergoing surgery directly were determined as HER-2 positive in this retrospective study. HER-2 overexpression was not associated with any clinical and pathological parameters. HER-2 might predict distant metastasis, but did not predict local recurrence. In term of clinical outcome, HER-2 positivity was significantly associated with poor 5-year DFS and 5-year OS. This study confirmed that HER-2 was really overexpressed in a part of patients with rectal cancers and might be used as a negative predictive marker. The results may support the trials to assess the efficacy of trastuzumab treating patients with HER-2 positive rectal cancer, which may be expected to improve the outcome of such patients.
